# Efficacy of physical therapy for prevention and treatment of postoperative lymphedema in breast cancer: a systematic review and meta-analysis

**DOI:** 10.3389/fonc.2026.1760801

**Published:** 2026-03-25

**Authors:** Xiaomin Sun, Yuyu Wei, XiaoXia Zhang, Xinqi Zhuang, Yin-Ping Zhang

**Affiliations:** 1 School of Nursing, Xi’an Jiaotong University Health Science Center, Xi’an, Shaanxi, China; 2 Department of nursing, Xi’an No.3 Hospital, the Affiliated Hospital of Northwest University, Xi’an, Shaanxi, China; 3 Cancer Center, West China Hospital, Sichuan University, Chengdu, Sichuan, China; 4 West China School of Nursing, Sichuan University, Chengdu, Sichuan, China; 5 Innovation Center of Nursing Research, Nursing Key Laboratory of Sichuan Province, Chengdu, Sichuan, China

**Keywords:** a systematic review, meta-analysis, postoperative lymphedema in breast cancer, prevention, treatment

## Abstract

**Background:**

Lymphedema represents a prevalent complication following breast cancer surgery, for which effective interventions can significantly reduce incidence rates.

**Aims:**

This systematic review and meta-analysis explored the clinical efficacy of different physical therapy interventions on breast cancer-related lymphedema (BCRL) in postoperative patients with breast cancer (BC).

**Methods:**

Comprehensive literature searches were conducted in eight databases: PubMed, Web of Science, Cochrane Library, ScienceDirect, ClinicalTrials.gov, CINAHL, Embase, and Scopus, from their inception until May 31, 2025. After study selection, meta-analyses were performed using Review Manager software (version 5.4). The primary outcome measures were the overall incidence of BCRL, the incidence of BCRL following specific physical interventions, and the incidence of BCRL after different intervention durations.

**Results:**

The initial search yielded 3566 publications. 31 studies were included after screening, comprising a total sample size of 4323 patients. Meta-analysis showed that physical intervention had no significant therapeutic (RR = 0.81, 95%CI 0.52 to 1.27, *P*>0.05) and preventive (RR = 0.72, 95%CI 0.50 to 1.02, *P*>0.05) effect on BCRL. Subgroup analyses further indicated that compared with 2-month, 3-month, 6-month,and 18-month intervention durations, physical intervention has a significant effect on BCRL. when administered for durations of 12 months (RR = 0.53, 95% CI 0.29 to 0.95, *P* < 0.05).

**Conclusion:**

Whether physical intervention is effective in the prevention and treatment of postoperative lymphedema in BC patients’ needs to be further explored, but there is a significant effect in the 12-month intervention subgroup. Therefore, we should pay more attention to the effect of physical intervention measures after enough time in clinical practice. The review protocol was registered prospectively in PROSPERO (Registration number: CRD42024615513).

**Systemetic Review Registration:**

https://www.crd.york.ac.uk/PROSPERO/myprospero, identifier CRD42024615513.

## Introduction

Breast cancer (BC) ranks among the most prevalent malignancies affecting women globally. According to World Health Organization (WHO) data, new BC cases are projected to rise from 2.3 million in 2022 to 3.0 million by 2040, indicating a persistent upward trend in annual incidence (1). However, advancements in early diagnosis and comprehensive BC treatment strategies have led to a gradual decline in mortality rates. The 5-year survival rate in developed countries can reach 87.4% (2). Consequently, managing postoperative complications in long-term BC survivors has become a critical challenge for rehabilitation. The studies suggest that early intervention can effectively prevent the development of postoperative complications (3).

Breast cancer-related lymphedema (BCRL) is a common postoperative complication of breast cancer patients. It is estimated that about 20%-30% (4, 5)of cases will occur, affecting about 4.7 million breast cancer survivors worldwide (6).BCRL not only causes limb swelling, pain, and restricted mobility but is also strongly associated with an increased risk of infection (e.g., cellulitis) and deterioration in mental health. The US Centers for Disease Control and Prevention (CDC) estimates that the average annual healthcare expenditure for BCRL patients is approximately $23,000 higher than for controls, primarily due to recurrent care, device utilization, and lost productivity (7). Furthermore, the significant financial burden may lead some patients to forgo standardized treatment, potentially exacerbating BCRL progression (8). BCRL represents a lifelong burden for breast cancer patients once it develops (9). Therefore, effective prevention and management of lymphedema are crucial components for improving the postoperative quality of life in BC survivors (10).

The clinical management and prevention of BCRL primarily rely on conservative therapies, predominantly various forms of physical interventions. These include techniques to promote lymphatic drainage (instrument-based or manual), resistance exercises, or dietary interventions (3, 11–13). Meta-analyses continue to synthesize the collective efficacy findings from different studies, with the majority demonstrating the effectiveness and safety of physical therapy for lymphedema. However, some meta-analyses have reported that physical interventions provides no benefit for lymphedema prevention or treatment. Two recent meta-analyses indicated that physical interventions can improve patients’ quality of life but show no significant effect on preventing or treating lymphedema (14, 15).

It has been identified that early assessment and intervention for lymphedema may enhance the effectiveness of physical interventions (16, 17). Given that lymphedema interventions typically require extended periods, usually involving more than three months, to demonstrate efficacy, no analysis has yet reported on the effects of different intervention durations. In addition, physical interventions can be applied with either a preventive or therapeutic intent for lymphedema. However, published meta-analyses have not distinctly differentiated between the preventive and therapeutic effects of physical interventions.

Taken together, this study aims to employ a systematic review approach to: 1) analyze the efficacy of physical interventions for BCRL: 2) investigate the effects of physical interventions specifically for the prevention versus the treatment of lymphedema: and 3) explore the impact of different intervention durations on the efficacy of physical interventions for BCRL. The findings are expected to provide multi-dimensional evidence on the efficacy of physical interventions for lymphedema, thereby offering a basis for clinicians to rationally apply these therapies according to individual patient characteristics.

## Methods

### Outcome index

In the present study primary outcome measures were the overall incidence rate of BCRL, the incidence rate of BCRL following specific physical interventions, and the incidence rate of BCRL after different intervention durations. Outcome Definition: BCRL was defined as limb swelling occurring at different time points after breast cancer surgery, characterized by an arm circumference difference of at least 2 cm or a limb volume difference of 200 ml (18).

This systematic review and meta-analysis were conducted according to the PRISMA 2020 statement (19).

### Data sources and search strategy

Comprehensive electronic searches were performed in the following eight databases from inception until May 31, 2025: PubMed, Web of Science, Cochrane Library, ScienceDirect, ClinicalTrials.gov, CINAHL, Embase, and Scopus. The search strategy was developed under the guidance of an evidence-based medicine specialist, utilizing a combination of Medical Subject Headings (MeSH) terms and free-text keywords. The core search strategy included:(“Breast Neoplasms”[Mesh] OR “Breast Cancer”[Title/Abstract]) AND (“Lymphedema/prevention & control”[Mesh] OR “Lymphedema/therapy”[Mesh] OR “Lymphedema - prevention and control”[Title/Abstract] OR “Lymphedema - therapy”[Title/Abstract]OR “Lymphedema Management”[Title/Abstract]OR “Lymphedema Treatment”[Title/Abstract]) AND(“Arm Lymphedema”[Title/Abstract] OR “swelling of the arm”[Title/Abstract] OR “Upper Extremity Lymphedema”[Title/Abstract])AND (“Exercise Therapy”[Mesh] OR “Compression Garments”[Mesh] OR “Manual Lymph Drainage”[Mesh] OR “Physical Therapy Modalities”[Mesh]). Additionally, the reference lists of all included studies and relevant reviews were manually screened. Registered clinical trials were also searched in CENTRAL and ClinicalTrials.gov to enhance the recall rate.

### Study eligibility criteria in this study

#### Inclusion criteria

1. Participants (P): The study population comprised surgically treated breast cancer patients, including those with or without the occurrence of lymphedema.
2. Intervention (I): Patients receiving physical therapy interventions aimed at preventing or treating BCRL (e.g., compression sleeves, manual lymphatic drainage, progressive resistance training, weight-lifting), the length of intervention is unlimited.
3. Comparator (C): Patients receiving non-physical therapy interventions, such as usual care (standard postoperative care without specific lymphedema prevention/therapy) or no intervention (wait-list control).
4. Outcomes (O): Studies reporting at least one of the following outcomes: BCRL incidence rate, incidence of BCRL after different intervention durations, or BCRL incidence rate comparing physical intervention to the comparator.
5. Study Design (S): Randomized Controlled Trials (RCTs), including both parallel-group and cluster-RCTs.

#### Excluded criteria

Studies meeting any of the following criteria were excluded:

1. Publication Type: Reviews, conference abstracts, letters to the editor, technical reports, or case reports.
2. Data Issues: Duplicate publications, studies where the full text was unavailable, or studies reporting incomplete outcome data.

### Data extract

Literature management was performed using EndNote X21 software. After duplicate removal, two investigators (SUN and WEI) independently conducted the literature screening, full-text review, and study selection according to the predefined inclusion and exclusion criteria. Data extraction and quality assessment were also performed independently by both reviewers. Any discrepancies arising during these processes were resolved through discussion with a third investigator (ZHANG).

The specific data items extracted from the included articles encompassed: author(s), publication year, country, sample sizes of intervention and control groups, randomization method, intervention details (type, duration, frequency), and outcome data (BCRL incidence rates, measurement timepoints).

### Quality evaluation

The methodological quality (risk of bias) of the included studies was assessed using the Cochrane Risk of Bias (RoB) tool, version 5.1.0 (20). This tool evaluates seven key domains: Random sequence generation (selection bias), allocation concealment (selection bias), blinding of participants and personnel (performance bias), blinding of outcome assessment (detection bias), incomplete outcome data (attrition bias), selective reporting (reporting bias), and other sources of bias. Each domain was judged as “Low risk”, “High risk”, or “Unclear risk” of bias based on the criteria outlined in the Cochrane Handbook.

### Statistical analyses

Statistical analyses were performed using Review Manager software (RevMan version 5.4). The risk ratio (RR) was used as the effect measure for dichotomous outcomes (e.g., BCRL incidence). For continuous outcomes, the mean difference (MD) was used as the effect measure. All effect estimates are reported with their corresponding 95% confidence intervals (CI). Heterogeneity among the included studies was assessed using Cochrane’s Q-test (Chi² test) and quantified using the I² statistic. The following thresholds guided the choice of analysis model: Low Heterogeneity (Fixed-effect model): I² < 50% and Q-test *P*-value ≥ 0.1. Substantial Heterogeneity (Further Investigation): I² ≥ 50% and/or Q-test *P*-value < 0.1. In this case, potential sources of heterogeneity (e.g., methodological or clinical differences) were explored using sensitivity and subgroup analyses. Random-effects model: If substantial heterogeneity persisted after exploration of potential sources, a random-effects model (DerSimonian and Laird method) was used for meta-analysis. A two-sided *P*-value < 0.05 was considered statistically significant for the overall effect estimate. Publication bias was assessed visually using funnel plots. Symmetry of the funnel plot was interpreted as suggesting the absence of significant publication bias. Other relevant data are described in a narrative way.

## Result

### Literature search

**Figure 1:**
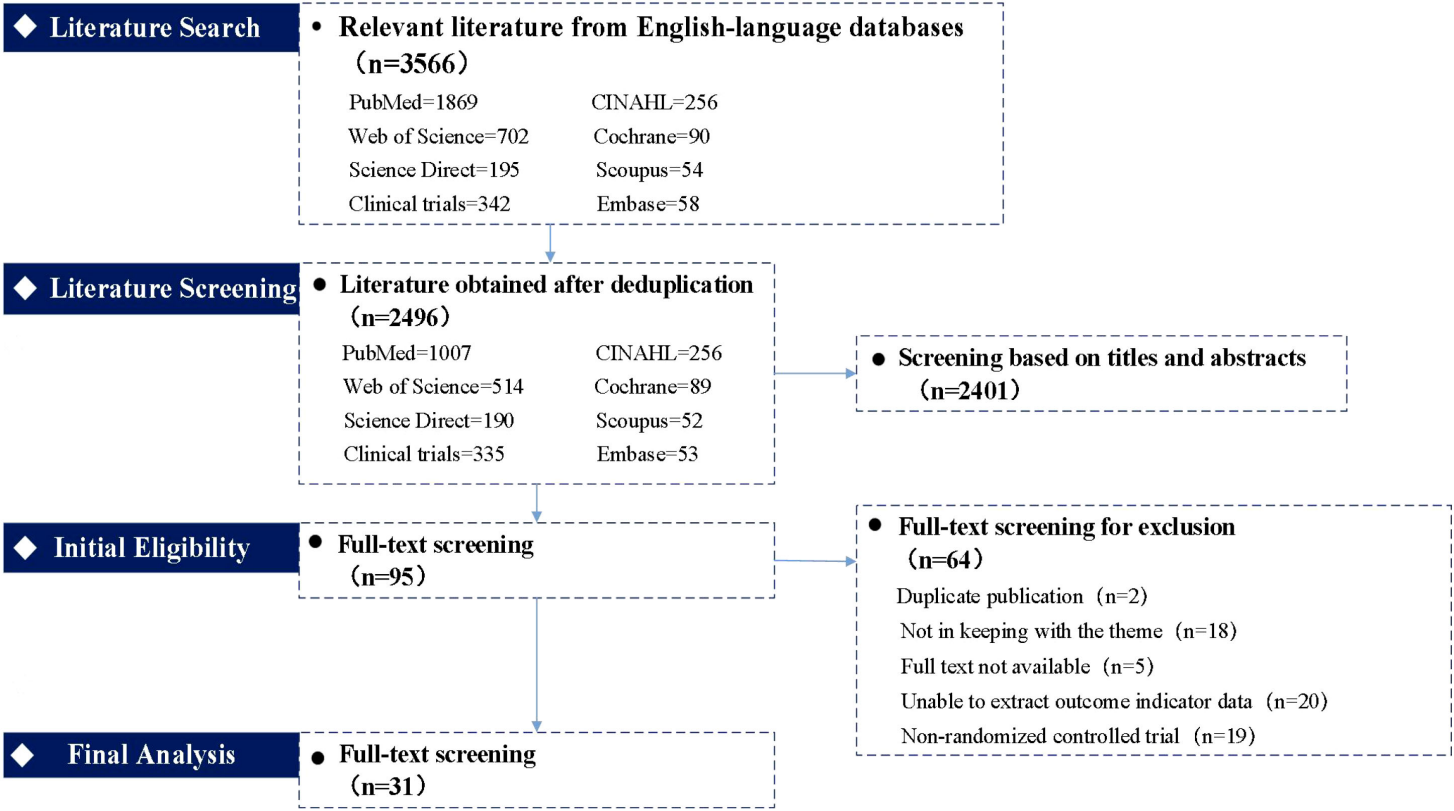
Literature screening flow chart.

The initial search yielded 3566 publications. After removing duplicates, 2496 articles remained. Screening of titles and abstracts resulted in 95 potentially relevant articles. Following full-text review, 31 studies were ultimately included in the analysis (21–52). The selection process is detailed in **Figure 1** (PRISMA flow diagram).

### Risk of bias assessment

**Figure 2:**
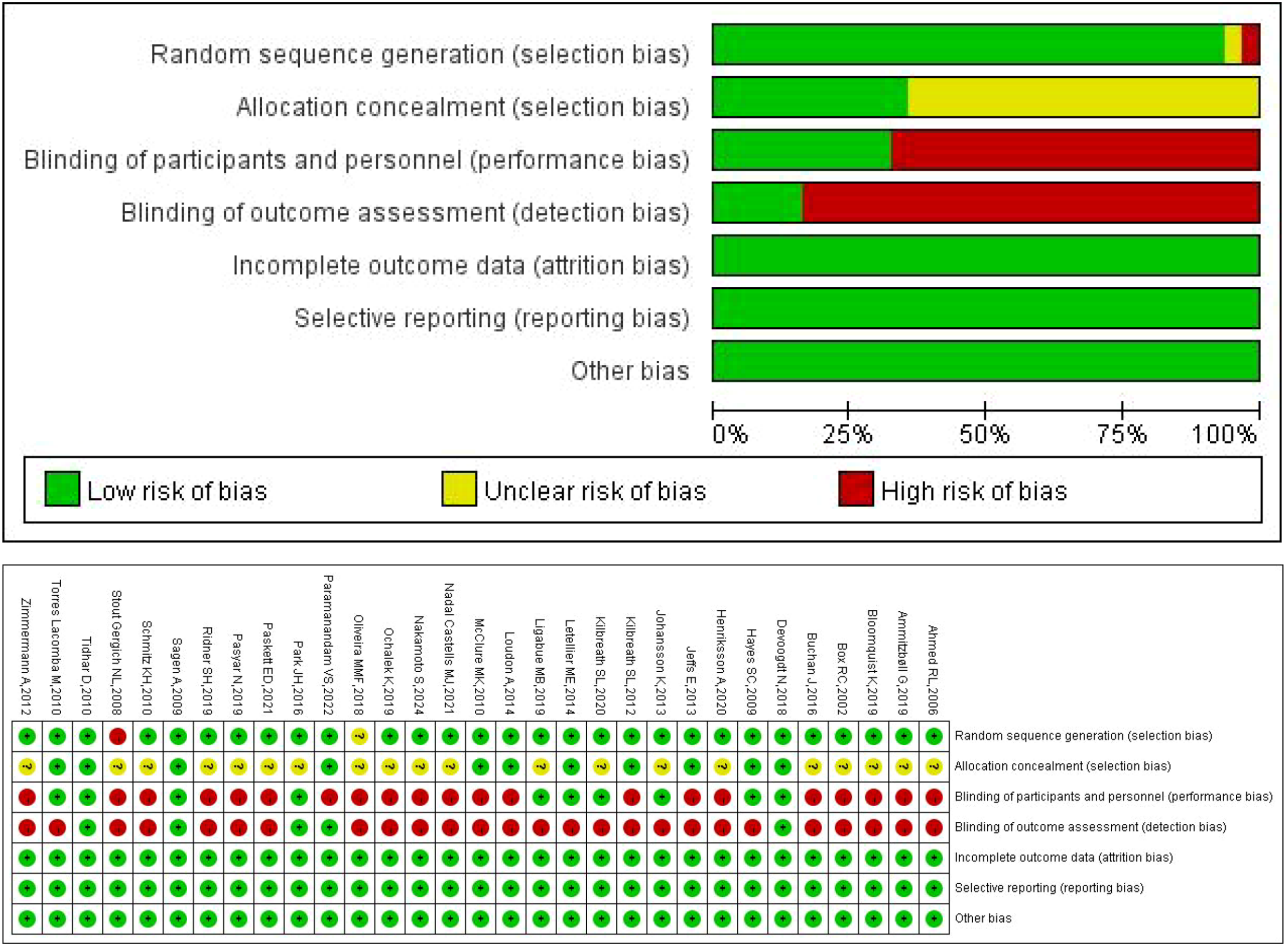
Literature quality evaluation chart.

The methodological quality of the included studies was assessed using the Cochrane Risk of Bias tool (version 5.1.0) (20). Among the 31 studies analyzed, the majority (n=20, 64.5%) were rated as having low or moderate risk of bias. The primary reason for a high-risk rating in the remaining studies was the lack of blinding of participants, personnel, and outcome assessors. The risk of bias summary is presented in **Figure 2**.

### Participant characteristics

The analysis included 31 two-arm RCTs, encompassing a total of 4323 breast cancer patients. The intervention groups comprised 1931 patients, while the control groups comprised 2392 patients. The included studies were predominantly conducted in the United States (n=7, 20.58%) and Australia (n=6, 17.64%), with other countries including Germany, Belgium, and Norway. The earliest study was published in 2002, with studies published within the last decade accounting for 50% (n=16) of the included literature.

### Intervention characteristics

Physical interventions primarily included: compression sleeves, manual lymphatic drainage (MLD), progressive resistance training, and weight-lifting. The main intervention durations analyzed were 2 months, 3 months, 6 months, 12 months, and 18 months.

Physical therapy interventions included: compression sleeves (6 studies, 17.64%, n=807 patients), MLD (4 studies, 11.76%, n=225 patients), resistance exercise (3 studies, 11.76%, n=259 patients), and other forms of physical exercise (17 studies, 54.83%, n=554 patients). Control groups primarily received usual care or no specific intervention. However, some studies (34.41%) (24–27, 30, 32, 36, 37, 39, 42) utilized a combination of physical interventions and usual care in their control arms. Details are provided in **Table 1**.

**Table 1:** Basic information included in the study.

Serial number	Included trials	Intervention measure		Time duration of intervention	Measurement method of outcome indicators	Prevention/ treatment
		Intervention group	Contrast group			
1	Box RC,2002 (Australia)	Early physical therapy, n=32	Routine care, n=33	1m	water displacement	T
2	Ahmed RL,2006 (United States)	Weight-lifting, n=23	Routine care, n=23	6m	Manual measurement of arm circumference	P
3	Stout Gergich NL,2008 (United States)	Pressurized sleeve, n=43	Routine care, n=43	1m	Manual measurement of arm circumference	P
4	Hayes SC,2009 (Australia)	Aerobic exercise+ Progressive resistance training, n=16	Routine care, n=16	3m	electric bio-impedance spectrum	P
5	Sagen A,2009 (Norway)	Unlimited activities+ Exercise, n=104	limitation of movement+ Routine care, n=100	6m	Manual measurement of arm circumference	P
6	McClure MK,2010 (United States)	Breast Rehabilitation Programme, n=16	One-to-one consultation, n=16	3m	electric bio-impedance spectrum	P
7	Tidhar D,2010 (Israel)	Hydrolymphatic therapy, n=16	Routine care, n=32	1m	water displacement	T
8	Torres Lacomba M,2010 (Spain)	Manual lymphatic drainage+ Progressive resistance training+Z, n=60	Routine care, n=60	12m	Manual measurement of arm circumference	T
9	Schmitz KH,2010 (United States)	Weight-lifting, n=72	Routine care, n=75	3m	water displacement	P
10	Zimmermann A,2012 (Germany)	Manual lymphatic drainage, n=33	Routine care, n=34	6m	water displacement	P
11	Kilbreath SL,2012 (Australia)	Progressive resistance training+ Exercise, n=81	Routine care, n=79	6m	electric bio-impedance spectrum	P
12	Jeffs E,2013 (United Kingdom)	Exercise, n=11	Routine care, n=12	6m	electric bio-impedance spectrum	P
13	Johansson K,2013 (Sweden)	Hydrolymphatic therapy, n=15	Routine care, n=14	2m	electric bio-impedance spectrum	T
14	Letellier ME,2014 (Canada)	Hydrolymphatic therapy, n=13	Exercise, n=12	3m	water displacement	P
15	Loudon A,2014 (Australia)	Yoga, n=15	Routine care, n=13	2m	electric bio-impedance spectrum	T
16	Buchan J,2016 (United States)	Progressive resistance training, n=21	Aerobic exercise, n=20	3m	electric bio-impedance spectrum	P
17	Park JH,2016 (Korea)	Complex exercise, n=32	Conventional inflatable therapy, n=31	1m	Manual measurement of arm circumference	P
18	Oliveira MMF,2018 (Brazil)	Manual lymphatic drainage, n=53	Exercise, n=53	1m	Manual measurement of arm circumference	T
19	Devoogdt N,2018 (Belgium)	Manual lymphatic drainage+C, n=79	Exercise, n=81	6m	water displacement	P
20	Pasyar N,2019 (Iran)	Yoga, n=20	Routine care, n=20	2m	water displacement	P
21	Ochalek K,2019 (Poland)	Pressurized sleeve, n=20	Routine care, n=21	12m	Manual measurement of arm circumference	P
22	Ligabue MB,2019 (Italy)	Early physical therapy, n=20	Routine care, n=21	6w	water displacement	T
23	Bloomquist K,2019 (Denmark)	Progressive resistance training, n=75	One-to-one consultation, n=78	3m	double energy x ray	P
24	Ammitzbøll G,2019 (Denmark)	Progressive resistance training, n=82	Routine care, n=76	5m	water displacement	P
25	Ridner SH,2019 (United States)	Pressurized sleeve, n=245	Routine care, n=263	2m	electric bio-impedance spectrum	P
26	Kilbreath SL,2020 (Australia)	Aerobic exercise+ Progressive resistance training, n=41	Routine care, n=47	3m	Manual measurement of arm circumference	T
27	Henriksson A,2020 (Sweden)	Equal intensity movement of planting land+high intensity exercise, n=90	Routine care, n=577	6m	Manual measurement of arm circumference	P
28	Nadal Castells MJ,2021 (Spain)	Routine care+ Pressurized sleeve, n=35	Routine care, n=35	3m	Manual measurement of arm circumference	P
29	Paskett ED,2021 (United States)	Health education+movement Pressurized sleeve, n=310	Health education n=244	18m	Manual measurement of arm circumference	P
30	Paramanandam VS,2022 (Australia)	Routine care+ Pressurized sleeve, n=154	Routine care, n=153	3m	electric bio-impedance spectrum	P
31	Nakamoto S,2024 (Japan)	Exercise+educational intervention, n=104	Routine care, n=111	4m	Manual measurement of arm circumference	P

### Objective 1: overall efficacy of interventions for BCRL

**Figure 3:**
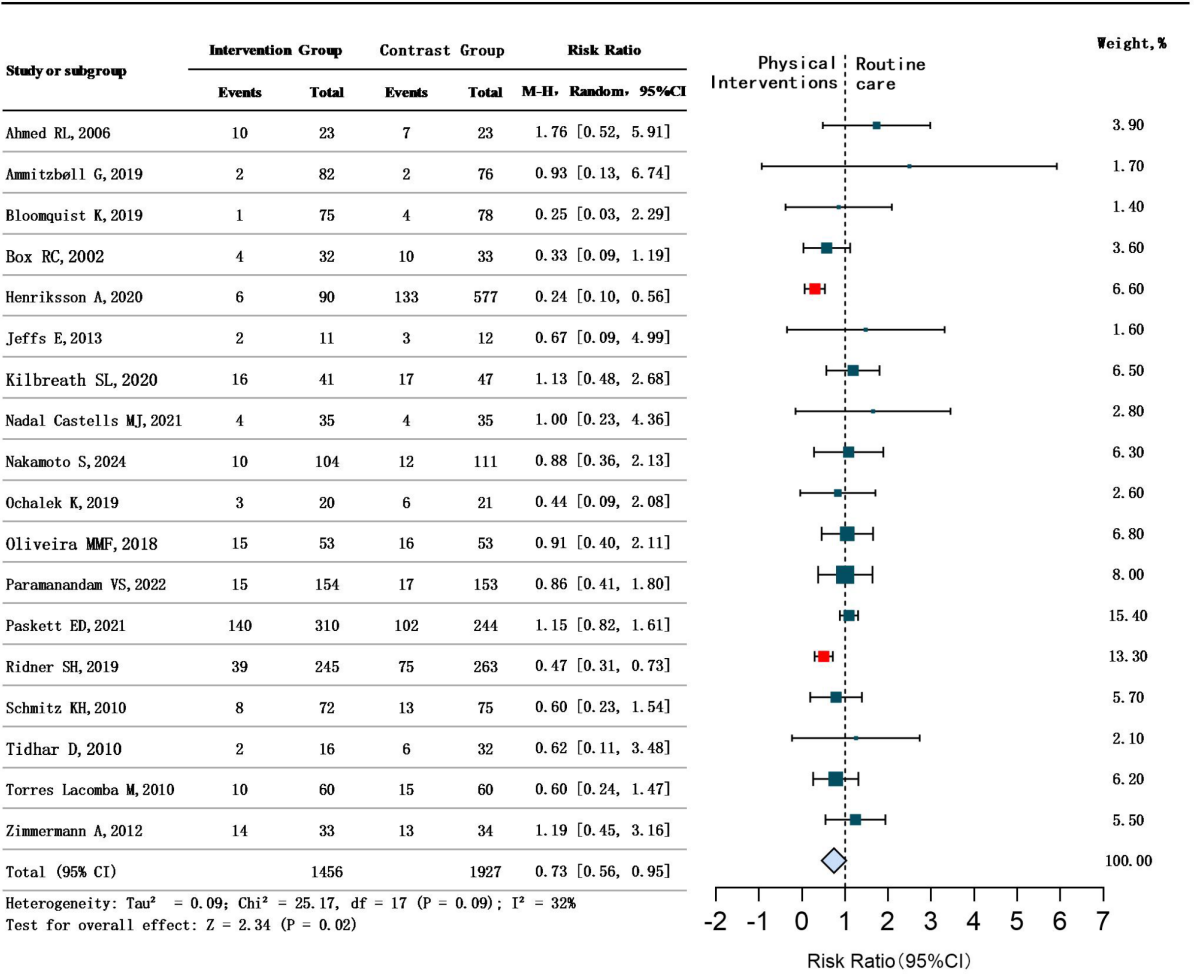
Overall therapeutic efficacy of physical interventions for BCRL.

All 31 studies evaluated the efficacy of physical interventions for BCRL. However, statistical analysis was performed only on the 18 studies that reported efficacy rates. Heterogeneity among these studies was low (*P* = 0.09,I²=32%), justifying the use of a random-effect model. The analysis revealed a significant overall benefit of physical interventions for BCRL (RR = 0.73, 95% CI 0.56 to 0.95, *P* < 0.05). There are statistical differences, suggesting that physical interventions have a significant effect on BCRL. See **Figure 3**.

### Objective 2: efficacy of physical interventions for prevention vs. treatment of BCRL

**Figure 4:**
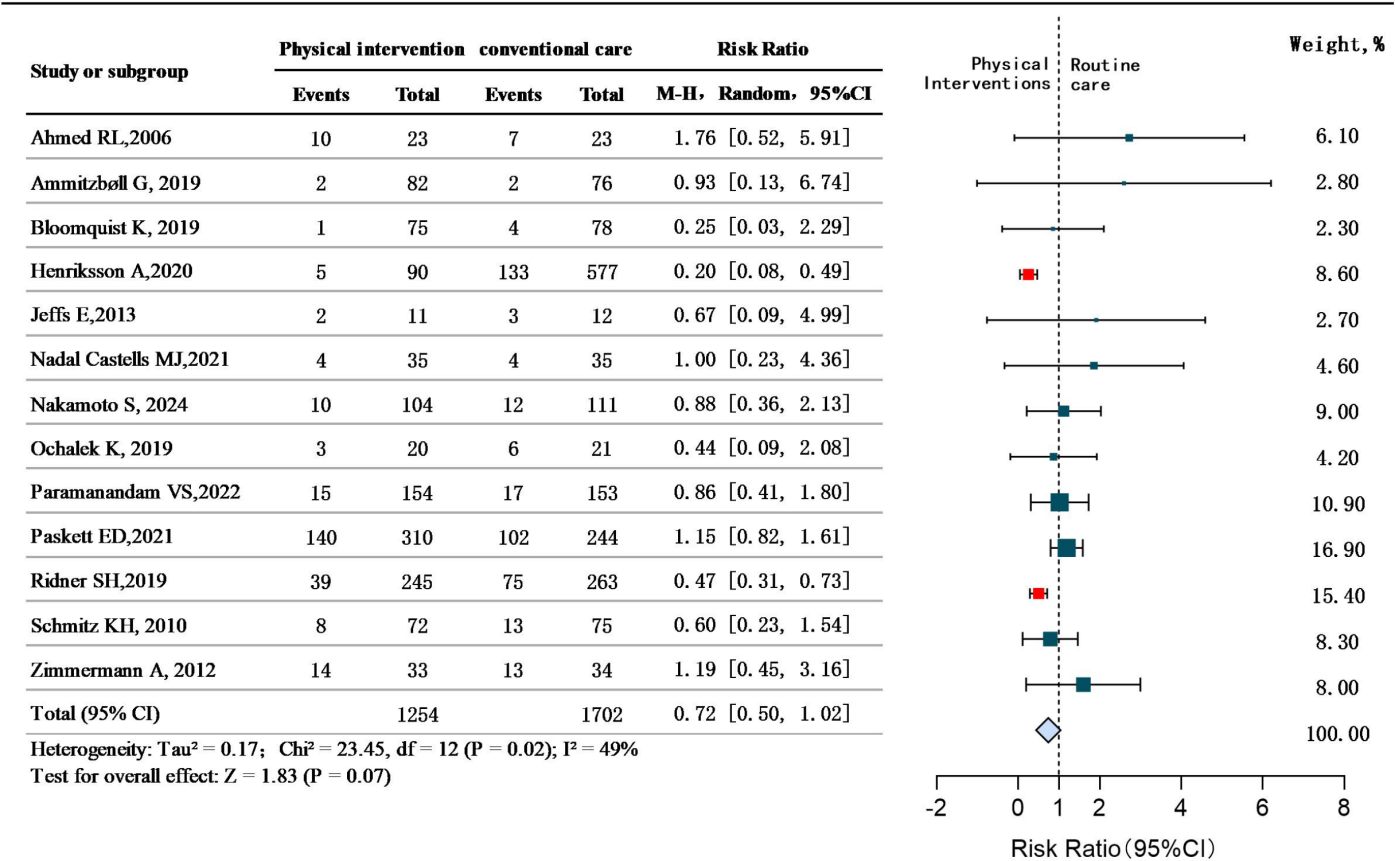
Preventive efficacy of physical interventions for BCRL.

Prevention: Thirteen studies (21–24, 28, 29, 33–35, 37, 38, 50, 51) specifically aimed to analyze the efficacy of physical interventions (e.g., weight-lifting, progressive resistance training, compression sleeves) for preventing BCRL. Heterogeneity was moderate but acceptable (*P* = 0.02,I²=49%), warranting a random-effect model. The results showed that physical intervention was not statistically significant in preventing BCRL(RR = 0.72, 95%CI 0.50 to 1.02, *P*>0.05).It is suggested that the effect of physical intervention on the prevention of BCRL needs to be further explored. See **Figure 4**.

**Figure 5:**
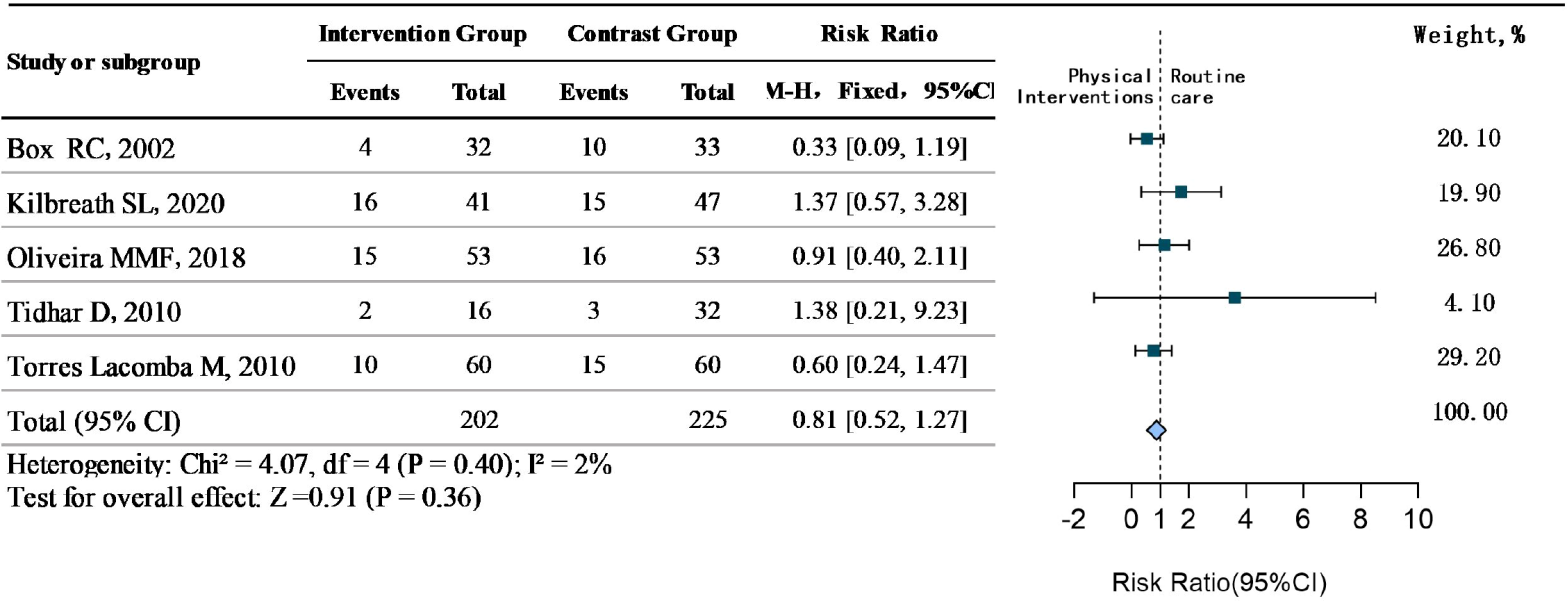
Treatment efficacy of physical interventions for BCRL.

Treatment: Five studies (41, 42, 45, 46, 49)specifically aimed to analyze the efficacy of physical interventions (e.g., MLD, aerobic exercise, resistance training) for treating established BCRL. Heterogeneity was negligible (*P* = 0.40,I²=2%), supporting the use of a fixed-effect model. The results showed that there was no statistical difference in physical interventions on lymphedema (RR = 0.81, 95%CI 0.52 to 1.27, *P*>0.05). It is suggested that the effect of physical interventions in the treatment of BCRL is limited, and it is necessary for professionals to choose appropriate intervention measures according to the actual situation of BC patients. See **Figure 5**.

### Objective 3: effect of intervention duration on BCRL outcomes

**Figure 6:**
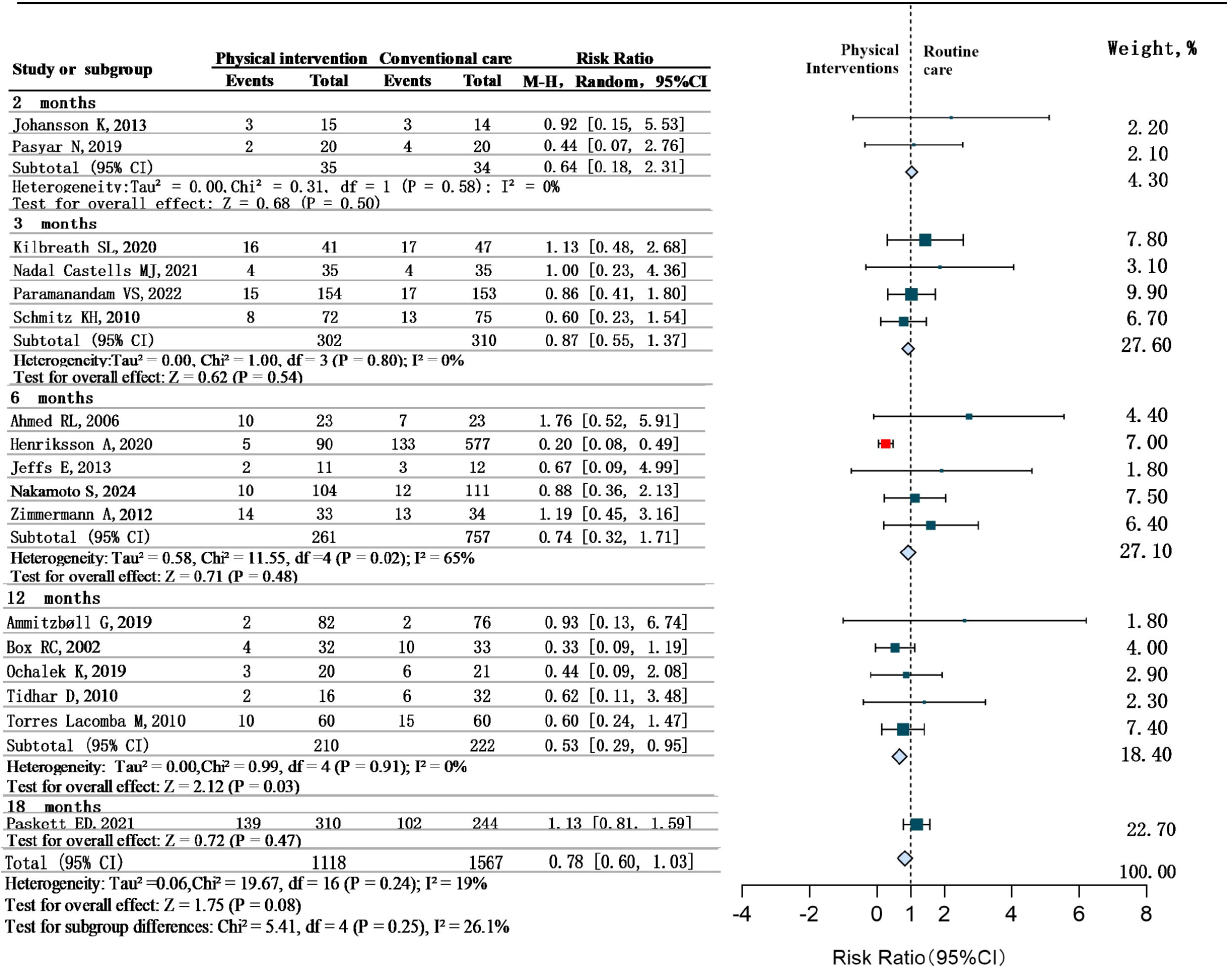
Time-stratified effects of physical interventions for BCRL.

Further subgroup analyses were conducted to examine the effect of different durations of physical therapy interventions on BCRL outcomes. Seventeen studies reporting intervention duration were included (21–23, 28, 29, 33–35, 37, 38, 41, 45, 46, 48–51). Reported durations varied widely, ranging from 2 to 18 months. Additionally, Appropriate effect models were adopted according to the values of the I² statistic. Studies were categorized into five duration-based subgroups: 2 months, 3 months, 6 months, 12 months, and 18 months. Heterogeneity within subgroups was as follows: 2-month group (*P* = 0.58, I²= 0%), 3-month group (*P* = 0.80, I²= 0%), 6-month group (*P* = 0.02, I²=65%), 12-month group (*P* = 0.91, I²=0%), and 18-month group (only one study). Analysis revealed differential effects based on duration: 2-month (RR = 0.64, 95% CI 0.18 to 2.31, *P*>0.05),3-month interventions (RR = 0.87, 95% CI 0.55 to 1.37, *P*>0.05) and 6-month interventions (RR = 0.74, 95%CI 0.32 to 1.71, *P*>0.05),showed non-significant prevention effects; 12-month interventions maintained significant effects (RR = 0.53, 95%CI 0.29 to 0.95, *P* < 0.05); 18-month analysis indicated non-significant therapeutic effect (RR = 1.13, 95%CI 0.81 to 1.59, *P*>0.05), The pooled effect across all five duration subgroups showed statistically significant differences (RR = 0.78, 95% CI 0.60 to 1.03, *P*>0.05). See **Figure 6**.

## Discussion

This meta-analysis included 31 RCTs to evaluate the therapeutic and preventive effects of physical intervention on lymphedema management. The overall combined analysis results of this study showed that physical intervention had a significant intervention effect on lymphedema after breast cancer surgery(RR = 0.73, 95% CI 0.56 to 0.95, *P* < 0.05).However, when subgroup analyses were performed according to intervention objective, neither the prevention subgroup (RR = 0.72, 95%CI 0.50 to 1.02, *P*>0.05)nor the treatment subgroup(RR = 0.81,95%CI 0.52 to 1.27, *P*>0.05) showed a statistically significant effect. In the subgroup analysis by intervention duration, only the 12-month intervention group exhibited a statistically significant difference(RR = 0.53,95%CI 0.29 to 0.95, *P* < 0.05), suggesting that a sustained intervention of adequate length may be critical for achieving a reliable therapeutic effect.

### The effectiveness of physical intervention in the prevention and treatment of BCRL remains to be further explored

In this study, physical intervention had no significant effect on reducing the incidence of lymphedema, contrary to the results of Bauman et al (53).Furthermore, recent studies have confirmed that exercise therapy, as a physical intervention, effectively reduces lymphedema occurrence (54, 55).Modalities such as resistance exercise and aerobic exercise enhance patients’ metabolic capacity, increase lymphatic vessel tolerance, and promote muscle contractility and lymphatic drainage. A study highlighted the positive role of resistance exercise specifically in preventing lymphedema after breast cancer surgery (56).This discrepancy between overall and subgroup findings may be attributed to differences in effect model selection. Recent meta-epidemiological evidence confirms that different pooling methods (fixed/random-effects models) can directly alter effect estimates and significance judgments, particularly when subgroup sample sizes are small and heterogeneity is substantial (57).Fixed-effects models tend to overestimate precision with narrower CIs, while random-effects models are more conservative with balanced weighting, often leading to reversed significance conclusions (58).When analyzed separately, the limited number of studies included in each subgroup made it difficult to identify a stable intervention effect. In contrast, pooling all studies increased the sample size and statistical power, thereby revealing an overall benefit of physical intervention.

Specifically, several major studies we included in the analysis showed that there was a significant difference in the sample size between the physical therapy group and the control group, which may introduce a bias in weight distribution during the merging process. Statistically, in both fixed-effects and random-effects models, studies with larger sample sizes are typically assigned greater weights; consequently, if the majority of these large-sample studies failed to demonstrate significant therapeutic benefits, the pooled results would inevitably lean toward a “null therapeutic effect” (59). Conversely, the relatively small sample size of the intervention group may have compromised the statistical power to detect significant differences in lymphedema outcomes, thereby obscuring the clinical meaningfulness of the observed improvements and the potential therapeutic efficacy of physical intervention (60).

Therefore, when explaining the results of this study, it is necessary to fully consider the impact of sample size differences. In the future, the clinical trials evaluating the effect of physical intervention on BCRL should give priority to the research design with balanced sample size and sufficient efficacy between groups, so as to provide more reliable evidence support for clinical practice.

### Differential effects of intervention duration on BCRL prevention and treatment

Sustained physical intervention for 12 months alone demonstrated significant advantages in the prevention and management of lymphoedema, while no statistically significant beneficial outcomes were observed with 2-, 3-, and 6-month intervention regimens. In contrast, the 18-month intervention failed to yield positive results, a conclusion constrained by both the limited number of included studies and poor patient adherence, thus lacking reliable clinical reference value for the time being. The specific therapeutic efficacy of the 12-month intervention was consistent with the findings of Kandasoglu H et al. (2024) (61). Furthermore, a multicenter observational study (2025) (62) enrolling 3144 patients with lymphoedema also confirmed that patients who maintained standardized physical intervention for a long duration (≥12 months) achieved significantly better oedema volume control rates and greater improvements in quality of life compared with those in the short-term intervention group. The core pathophysiological mechanism of lymphoedema lies in impaired lymphatic drainage following lymphatic system injury, and the regeneration of lymphatic vessels and establishment of collateral circulation are inherently long-term, sustained processes. Studies have confirmed that the complete cycle of proliferation, migration, and functional maturation of lymphatic endothelial cells takes 10–12 months (63).

Although the early postoperative period (1–6 months) represents a phase of lymphatic vessel regeneration, newly formed lymphatic vessels exhibit a fragile structure and immature function, rendering stable lymphatic fluid drainage unattainable (64). The null result of the 6-month intervention in the present study further validates the notion that short-term initiation of regeneration is insufficient to reverse the pathological state, only when intervention is sustained for 12 months, allowing newly formed lymphatic vessels to complete structural remodeling and functional maturation, can strengthened collateral circulation significantly reduce interstitial fluid accumulation and achieve effective prevention and control of lymphoedema (64). The null results of the 2-, 3-, and 6-month interventions in the present study were highly consistent with those of a recent investigation. A state-of-the-art systematic review (2025) (65) indicated that short-term exercise intervention (≤3 months) only alleviated subjective symptoms such as limb heaviness and tightness but failed to reduce oedema volume significantly; 6-month exercise intervention improved lymphatic drainage velocity yet did not reach the clinically effective threshold; and only 12 months of sustained exercise intervention enabled the establishment of long-term adaptability of lymphatic vasomotor function, leading to a significant reduction in oedema volume (a mean decrease of 18.9%) (65). This conclusion was in full accordance with the findings of the present study.

The absence of beneficial outcomes in the 18-month intervention of the present study does not indicate an inherent lack of clinical value of the 18-month intervention regimen itself, but is instead attributable to the combined effects of the limited number of included studies and poor patient adherence. First, the present study incorporated only one relevant investigation focusing on 18-month physical intervention, and both the sample size and the number of included studies failed to meet the basic requirements for statistical testing, which is prone to inducing statistical bias and thus generating misleading conclusions. Second, the included study explicitly stated that only 30% of patients completed the entire 18-month intervention cycle, and this extremely low adherence rate (30%) severely attenuated the actual intensity and sustained therapeutic effect of the intervention. A large body of existing research has confirmed a significant positive correlation between the efficacy of physical intervention and patient adherence: when the daily average duration of compression garment wear is less than 8 hours or the adherence rate to exercise intervention is below 60%, the expected clinical outcomes are difficult to achieve even with prolonged intervention duration (50, 66). The 30% adherence rate in this 18-month study was far below the clinically effective threshold, and its null result is more likely a false negative caused by insufficient adherence rather than an issue with the intervention duration per se.

From a pathophysiological perspective, 12 months of physical intervention is sufficient to achieve lymphatic vessel regeneration and maturation, fibrosis reversal, and stable improvements in lymphatic dynamics. As a longer intervention regimen, 18 months should theoretically further maintain or even enhance the therapeutic efficacy of physical intervention, rather than leading to efficacy attenuation. Therefore, the null result of the 18-month intervention in the present study is essentially the combined outcome of study design flaws (insufficient sample size) and extremely low patient adherence, and thus holds no clinical reference significance. Future research should incorporate more large-sample, high-quality clinical studies on 18-month physical intervention and optimize intervention regimens to improve long-term patient adherence, thereby clarifying the clinical value of physical intervention exceeding 12 months in the management of lymphoedema.

## Conclusions

The subgroup analysis of this study showed that the two groups did not reach statistical significance when they were divided into prevention and treatment subgroups according to the purpose of intervention. However, when subgroup analysis was performed according to the duration of intervention, 12 months of intervention could significantly improve the outcome of lymphedema. This result suggests that the effect of physical intervention does not depend on “whether the purpose is prevention or treatment”, but on “whether the intervention is long enough and sustainable enough “.Because the prevention and treatment subgroups included interventions of different durations, the heterogeneity was high, so no significant effect was shown in the separate analysis. The 12-month long-term intervention group was more concentrated and the effect was more stable, so it could reflect statistical differences.

Therefore, for high-risk patients with lymphedema after breast cancer surgery, it is recommended to initiate preventive interventions (such as pressure sleeves or progressive resistance exercises) for ≥ 12 months immediately after surgery. The duration of individualized intervention was adjusted for patients with edema, such as comprehensive detumescence therapy (CDT) for at least 12 months. However, the existing evidence still has limitations. At present, for different physical interventions, due to the intensity and frequency of intervention and other aspects of the research conclusions cannot form a unified conclusion (67). it is recommended to develop a scientific exercise plan based on the individual situation of the patient and under the guidance of professionals (18).At the same time, attention should be paid to compliance management to ensure the full play of the intervention effect. For the potential value of the 18-month intervention, it is necessary to explore the feasibility of the intervention program after more high-quality studies are confirmed.

Most notably, we did not perform a pooled analysis of the variations in surgical approaches. The baseline risk of developing breast cancer-related lymphedema is associated with the type and extent of axillary surgery. Clinicians should formulate stratified physical therapy strategies based on the traumatic degree of surgical intervention and individual patient risk (68, 69). Literature review found that for low-risk patients undergoing sentinel lymph node biopsy, a 12-month physical therapy regimen is unnecessary, and individualized streamlined intervention is preferable. SLNB patients exhibit rapid recovery of lymphatic function postoperatively, with the majority regaining preoperative functional levels within 4–6 weeks; thus, long-term intensive intervention yields no additional benefits (68, 70). However, for high-risk patients undergoing axillary lymph node dissection (ALND): a 12-month regimen is mandatory, and whole-course management is critical. The progression of lymphedema in ALND patients is insidious, with some developing clinical symptoms only at 6 months postoperatively. Furthermore, once lymphedema progresses to a moderate stage (circumferential difference of 3–6 cm), reversal becomes significantly more challenging (71). Therefore, adherence to the 12-month regimen is directly associated with long-term quality of life and should not be shortened or simplified (68, 69).

Consequently, the observed differences in intervention efficacy may be partially attributable to underlying variations in surgical techniques. Future studies should focus on patient-level analyses that stratify outcomes based on the specific surgical procedures used.

## Data Availability

The original contributions presented in the study are included in the article/supplementary material. Further inquiries can be directed to the corresponding author.
